# A review of actinic keratosis, skin field cancerisation and the efficacy of topical therapies

**DOI:** 10.1111/ajd.13447

**Published:** 2020-08-25

**Authors:** Robert Sinclair, Christopher Baker, Lynda Spelman, Madeleine Supranowicz, Beth MacMahon

**Affiliations:** ^1^ Specialist Connect Services Brisbane Queensland Australia; ^2^ St Vincent’s Hospital Melbourne Victoria Australia

**Keywords:** actinic keratosis, non‐melanoma skin cancer, skin field cancerisation, skin neoplasms, topical therapy

## Abstract

While a wide range of treatments exist for actinic keratosis and skin field cancerisation, the long‐term benefits of the most common topical therapies are poorly defined. This report reviews the efficacy of the most commonly used topical therapies to treat regional or field lesions. Limited clinical and histopathological data are available on clearance rates at 12 months post‐treatment for the most commonly used agents, with varied outcome measures making any comparison difficult. In general, total field clearance rates at 12 months are suboptimal for the most commonly employed agents. Given the increasing incidence of actinic keratosis and skin field cancerisation due to an ageing population, further research into the efficacy of therapies is critical to guide treatment choice.

## Introduction

Skin field cancerisation describes areas of solar damage involving actinic keratosis, the clinical signs of photoageing, and variable numbers and types of skin cancers. These areas also commonly have a history of previous skin cancers and are prone to develop new malignancies, including nonmelanoma skin cancer, melanoma and rarely Merkel cell carcinoma. Current topical treatment options include 5‐fluorouracil (5‐FU), imiquimod, ingenol mebutate, diclofenac sodium and aminolevulinic acid photodynamic therapy (PDT). The goal of these therapies is to reduce the numbers of actinic keratosis and to prevent future cancer development. However, despite the burden and prevalence of actinic keratosis and skin field cancerisation, there is little understanding of treatment outcomes, as measured by recurrence or cancer prevention.

To address this point, we conducted a literature review to elucidate the effectiveness of commonly used topical therapies. The PubMed/MEDLINE database (https://www.ncbi.nlm.nih.gov/pubmed/), Cochrane Library and Web of Science were searched from January 1962 to February 2019 to identify relevant English language publications using the following specific search terms: ‘(skin field cancerisation OR skin field cancer) AND (actinic keratosis OR solar keratosis) AND (therapy OR topical OR 5‐fluorouracil OR imiquimod OR ingenol mebutate OR diclofenac sodium OR aminolevulinic acid OR Metvix OR photodynamic therapy OR light therapy) OR (efficacy of topical therapy)’. The retrieved titles and abstracts were screened for review on the efficacy for actinic keratosis and skin field cancerisation. For the purpose of this review, data from the face and scalp were selected for inclusion, and the treatment regimens analysed approximated the common treatment schedules. This review also provides an overview on actinic keratosis, skin field cancerisation, current methods of grading skin field cancerisation and limitations of the studies analysed.

### Actinic keratosis and skin field cancerisation

Actinic keratosis is a keratotic lesion occurring on chronically light‐exposed adult skin presenting as a dry, rough, sometimes pigmented lesion of variable thickness and size.^1^ It is the principal visible marker of solar damage for both clinical and research purposes^1^, ^2^ and remains the most reliable indicator for sun‐related skin cancers, especially squamous cell carcinoma (SCC).^1^, ^3^ Most research supports a greater risk of progression of thicker actinic keratosis to SCC,^4^, ^5^ and most topical treatments, except for 5‐FU, specifically exclude their use for these lesions.^1^, ^6^, ^7^, ^8^ There are three directions that actinic keratosis can evolve – spontaneous disappearance, persistence without progression to invasive SCC and progression to invasive SCC.^1^, ^9^ Spontaneous remission of actinic keratosis has been estimated to average around 25% per annum, but has been variably reported as between 15 and 60% per annum in different studies.^1^, ^3^, ^5^, ^10^, ^11^ Of lesions that visibly regress, between 15 and 53% subsequently recur.^10^ This high turnover rate complicates and confounds attempts to accurately track individual lesions for study purposes.^1^, ^12^, ^13^

Actinic keratosis is also a visible hallmark of skin field cancerisation and often helps to define its extent.^1^, ^11^, ^14^ As a concept, skin field cancerisation suggests that apparently ‘normal’ skin around an actinic keratosis already has genetic changes associated with carcinogenesis and often along multiple tumour development pathways.^5^, ^15^ Although cryotherapy remains the gold standard for individual lesions with reported cure rates between 75 and 95%,^8^ multiple studies support the need to treat cancer fields rather than individual actinic keratosis in the hope of reducing future tumour development.^1^, ^5^, ^11^, ^16^

### Current methods and limitations of grading assessments

Most historical studies have graded field damage on simple actinic keratosis counts, but measured in a variety of ways.^9^, ^17^ Efficacy measurements have most commonly employed complete clearance, reduction in lesion count and sustained clearance rates,^9^ but with most studies still relying exclusively on actinic keratosis counts.^11^ Others have combined counts with features such as degree of hyperkeratosis or severity of individual actinic keratosis using the Olsen scale as the only formalised clinical tool available.^18^ This can be combined with the Roewert–Hubert histological classification scale to assess individual, isolated actinic keratosis.^19^ However, one study found that only about half of the investigated lesions matched in terms of grading severity between the two scales, emphasising the difficulty of accurately grading individual actinic keratosis by clinical inspection.^5^, ^20^

Actinic keratosis numbers alone have been criticised for unreliability of grading because of significant differences in investigator counts and for being nonreproducible with significant count variations over time.^7^, ^11^, ^17^, ^21^, ^22^ The latter compounds the difficulties of comparing studies with varying post‐treatment assessment periods. Moreover, reductions in size or thickness of lesions as a result of treatment were not usually accounted for and may result in a false‐negative effect.^17^ Criticism of some studies has been made on the basis of limited size of test areas,^17^ low patient numbers^3^ and the arbitrary selection of test sites as it is common to choose only the most visibly damaged part of an anatomic area for scrutiny.^1^ The lack of a good field cancerisation grading scale has also likely contributed to poor assessment of the extent of solar damage prior to treatment and for consistent reappraisals after treatment.^17^

### Limitations of efficacy studies on common topical therapies

Numerous studies have tested the efficacy of common topical therapies for actinic keratosis. Despite this, there is a significant lack of high‐quality randomised controlled trials and the durability of outcomes for multiple therapies has not been established.^23^ In particular, comparisons between topical therapies have been hampered by different outcome measures, such as clearance of target lesions only,^24^ percentage change in actinic keratosis numbers,^25^ percentage change in area of actinic keratosis coverage and number of patients with partial (>75%) or complete (100%) field clearance.^9^, ^17^ Of the reported indices, complete field clearance appears less vulnerable to the high turnover of visible lesions and the emergence of new lesions within a field.^11^ However, complete field clearance at 12 months is a difficult and possibly unreasonable standard to achieve, and as a consequence, partial field clearance (>75%) is commonly used to provide a more meaningful comparison of therapeutic efficacy.^17^, ^26^ Data for FDA‐approved agents focus primarily on mean and/or median percentages of both complete (100%) and partial (>75%) field clearance, with evaluation of these responses restricted within a designated target area.^26^ For example, the most recent approval process for ingenol mebutate was based on complete and partial field clearance of 42.2% and 63.9% at Day 57, respectively.^26^, ^27^ Complete clearance rates at 12 months were termed ‘sustained clearance’ and were based on those fields clear at Day 57 that maintained complete clearance at 12 months. For ingenol mebutate, this was 46.1% for actinic keratosis of the face and scalp.^21^, ^26^

A major review of topical 5‐FU identified only 13 out of 29 randomised controlled trials with meaningful data for analysis. Of these, only three reported on patients at 12 months.^17^ Overall, very few studies report on follow‐up assessments beyond a few months, despite the significant recurrence rate of actinic keratosis as early as 12 months post‐treatment.^7^, ^8^, ^17^ Further, virtually no studies reported on the baseline severity of field cancerisation before treatment^28^ other than to exclude hyperkeratotic^7^, ^29^ and sometimes atypical actinic keratosis.^22^ Efficacy has also been linked to course duration for 5‐FU, imiquimod and diclofenac sodium but very few studies account for patient compliance with prescribed protocols^28^, highlighting a failure to account for ‘real‐life’ effectiveness of therapies.^1^, ^30^, ^31^

### Efficacy of common topical therapies for actinic keratosis and skin field cancerisation

In this review, the percentage reduction of actinic keratosis counts measured at 3–6 and 12 months was compared to baseline counts (Table 1), along with the percentage recurrence rates of actinic keratosis at 12 months that were initially clear at 2–3 months (Table 2). Table 3 also collates the percentages of treated fields that achieved total clearance of actinic keratosis at 3 and 12 months for each of the therapies examined. Use of complete field clearance was included as it avoids the pitfalls of trying to record individual lesions in the ever‐changing landscape of recurring and regressing actinic keratosis within the field.^21^ For some treatments, no direct 12‐month clearance counts relative to baseline were available. Of the studies that reported this outcome, the best was for 5% 5‐FU and imiquimod. As shown in Table 1, sustained clearance is also reported. This refers to a percentage recurrence rate at 12 months for those lesions that were documented as cleared at 3 months. It can be calculated by the product of the absolute clearance at 3 months by the subsequent recurrence of cleared lesions at 12 months, when reported from the same data set in a single study.^21^, ^26^

**Table 1:** Percentage reduction in actinic keratosis counts reported at 3–6 and 12 months

Treatment	% cleared at 3–6 months	Sustained clearance rates	% cleared at 12 months
5‐FU 5%	**94%** ^40^ **, 82%** ^41^ **, 79.2%** ^32^ **, 73%** ^42^ **, 59%** ^43^ **;** *87.8%* ^44^,*79.5%* ^17^	**54%** ^8^	**76.6%** ^32^ **, 54%** ^42^ **;** *42.9%* ^17^
Imiquimod 5%	**86.6%** ^45^ **, 86.5%** ^46^ **, 83.3%** ^47^ **, 81.8%** ^48^ **, 75.7%** ^49^ **, 74.4%** ^50^ **, 73%** ^8^ **, 66%** ^40^ **;** *65.9%* ^17^,*55%* ^1^	**73%** ^8^ **, 61%** ^50^ **;** *61–82.6%* ^1^	53.3% ^8^ , 45.3% ^50^ , *33.5–45.4%* ^1^
Diclofenac 3%	**51.5%** ^41^ **;** *89%* ^17^,*39.1%* ^51^	*79%* ^1^	*40.7%* ^1^
Ingenol mebutate	**81.3%** ^30^	**87.2%** ^21^ **, 46.1%** ^37^	**46.1%** ^21^ **;** *37.4%* ^1^ *;* 70.9% ^21^, ^30^
ALA‐PDT	**91%** ^52^ **, 89.5%** ^6^ **, 89%** ^24^ **, 86.9%** ^53^ **, 86.2%** ^54^ **;** *80%* ^37^,*80%* ^17^	**59.2%** ^37^, ^55^ **;** *83%* ^1^	**74.5%** ^25^

Primary study results (direct data) are in **bold**; review study estimates are in *italics*; and calculated percentages are underlined. Sustained clearance rates based on complete field clearance of actinic keratosis at 3 months that maintained complete clearance at 12 months.

**Table 2:** Percentage recurrence rates of actinic keratosis at 12 months that were initially clear with topical therapy at 2–3 months

Treatment	12‐month recurrence rates
5‐FU	**46%** ^8^ **;** *65%* ^1^
Imiquimod	**39%** ^50^ **, 27%** ^8^ **;** *17.4–39%* ^1^,*20.9%* ^56^
Diclofenac	***21%*** ^57^
Ingenol mebutate	*54%* ^1^,*12.8%* ^56^
ALA‐PDT	*40.8%* ^37^,*17%* ^1^

Primary study results (direct data) are in **bold**; review study estimates are in *italics*.

**Table 3:** Percentage of patients who achieved total field clearance of actinic keratosis at 3 and 12 months

Treatment	3 months	12 months
5‐FU	**96%** ^8^ **, 84%** ^40^ **, 43%** ^43^ **, 38%** ^42^ **;** *62.5%* ^44^,*52%* ^58^,*49* ^17^,*43–96%* ^1^	33%^8^ *33%* ^17^,*28%–62%* ^1^,*22%* ^42^
Imiquimod	**85%** ^8^ **, 68.9%** ^59^ **, 57.1%** ^60^ **, 55%** ^49^ **, 53.7%** ^50^ **, 50%** ^61^ **, 48.3%** ^45^ **, 45.1%** ^47^ **, 35.6%** ^48^ **, 35.6%** ^56^ **, 30.2%** ^46^ **, 24%** ^40^ **;** *84%* ^17^,*70%* ^58^,*50%* ^37^,*37.1%* ^9^,*26.8–55%* ^1^	**73%** ^8^ **;** 32.7% ^50^
Diclofenac	**58%** ^57^ **, 47%** ^62^ **;** *31.3%* ^9^	**24%** ^63^
Ingenol mebutate	**42.4%** ^37^ **, 42.2%** ^27^ **;** *42%* ^1^, *33.1%* ^9^	*18.5%* ^1^ *;* 22.8% ^27^
ALA‐PDT	**69%** ^64^, **59.2%** ^54^ **, 59%** ^65^ **;** *59–91%* ^1^,*58%* ^9^,	*49–75.5%* ^1^ *;* 34.8% ^37^, ^65^
Cryotherapy (only visible lesions treated)	Not available	**4%** ^8^, **3.3%** ^46^

Primary study results (direct data) are in **bold**; review study estimates are in *italics*; and calculated percentages are underlined.

Field therapies aim to reduce actinic keratosis for several reasons. There is undoubted benefit in lessening skin irritability and improving appearance, but the primary goal has always been to reduce the incidence of new sun‐related cancers.^3^, ^32^ Until recently, virtually no studies existed that demonstrated a reduction in skin cancer incidence following the use of the commonly available topical therapies for actinic keratosis.^1^, ^9^, ^11^, ^28^ A study following 932 participants for four years after 5‐FU found an incidence of 299 new basal cell carcinomas (BCC; 95 in year one) and 108 new SCCs (25 in year one) within the treated fields.^33^ The emergence of new SCC, but not BCC, was reduced in the first year compared to controls, but thereafter cancer rates returned to those of the untreated group with no discernible ongoing prophylactic benefit.^33^ Another recent retrospective study followed patients for five years after the treatment of field cancerisation on the face with 5‐FU and imiquimod. They identified 1,408 new cancers from the 5700 patients treated, but found no difference in efficacy between 5‐FU and imiquimod. Unfortunately, no comparable control group was reported.^34^

## Discussion

All the common topical therapies reviewed provided some short‐term reduction in actinic keratosis with 5% 5‐FU and PDT being the most effective formulations for short‐term clearance and ingenol mebutate being marginally less effective. Overall clearance at 12 months was marginally better for PDT compared to other agents but all had significant recurrence in a field of cancerisation, with approximately 50% of the baseline numbers evident at one year post‐treatment. Treatment combinations appear to improve efficacy compared to monotherapies but at significant additional cost.^35^ Of note, this review is limited to face and scalp, and significantly poorer outcomes are routinely reported for upper limbs.^36^

The natural history of actinic keratosis demonstrates a very high turnover, with large numbers developing, regressing and recurring over time.^1^ This labile nature emphasises the need for future studies to more accurately trace the evolution of all lesions included at baseline, by both number and specific location. The potential inherent inaccuracy of relying solely on lesion counts is based on this inability to distinguish recurrent from new lesions.^14^, ^17^, ^37^ It would seem essential that all new trials ensure outcomes are related more precisely to baseline solar damage^17^, ^38^ and follow‐up periods of at least 12 months should be routinely reported.^3^, ^39^

In summary, current topical therapies provide good short‐term clearance of actinic keratosis but significantly lower clearance rates by 12 months. The short follow‐up periods reported for many studies may reflect a desire to maximise apparent benefits while satisfying minimal regulator review requirements. The FDA approvals process have had to accommodate significant differences in study protocols.^26^ The acceptance of such short follow‐up data may have the downside of not providing real‐world evidence. More importantly, if the rationale behind field therapy is to reduce long‐term cancer incidence, the available evidence for most agents supports a short‐ to medium‐term benefit only and hence the need for ongoing surveillance with repeated treatments.
